# Light Respiratory Processes and Gross Photosynthesis in Two Scleractinian Corals

**DOI:** 10.1371/journal.pone.0110814

**Published:** 2014-10-31

**Authors:** Verena Schrameyer, Daniel Wangpraseurt, Ross Hill, Michael Kühl, Anthony W. D. Larkum, Peter J. Ralph

**Affiliations:** 1 Plant Functional Biology and Climate Change Cluster, School of the Environment, University of Technology, Sydney, Ultimo, New South Wales, Australia; 2 Centre for Marine Bio-Innovation and Sydney Institute of Marine Science, School of Biological, Earth and Environmental Sciences, The University of New South Wales, Sydney, New South Wales, Australia; 3 Marine Biological Section, Department of Biology, University of Copenhagen, Helsingør, Denmark; 4 Singapore Centre on Environmental Life Sciences Engineering, School of Biological Sciences, Nanyang Technological University, Singapore, Singapore; Auckland University of Technology, New Zealand

## Abstract

The light dependency of respiratory activity of two scleractinian corals was examined using O_2_ microsensors and CO_2_ exchange measurements. Light respiration increased strongly but asymptotically with elevated irradiance in both species. Light respiration in *Pocillopora damicornis* was higher than in *Pavona decussata* under low irradiance, indicating species-specific differences in light-dependent metabolic processes. Overall, the coral *P. decussata* exhibited higher CO_2_ uptake rates than *P. damicornis* over the experimental irradiance range. *P. decussata* also harboured twice as many algal symbionts and higher total protein biomass compared to *P. damicornis*, possibly resulting in self-shading of the symbionts and/or changes in host tissue specific light distribution. Differences in light respiration and CO_2_ availability could be due to host-specific characteristics that modulate the symbiont microenvironment, its photosynthesis, and hence the overall performance of the coral holobiont.

## Introduction

The success of scleractinian corals in oligotrophic tropical waters is based on the endosymbiosis between the coral host and single-celled microalgae, i.e., dinoflagellates in the genus *Symbiodinium* that reside within the host's endodermal cells. The algal symbionts translocate up to 95% of their photosynthetically fixed carbon (C) to the coral host under optimal conditions [1], whilst the algal symbionts receive nutrients and shelter from the host [2], [3]. There is considerable genotypic variation within the *Symbiodinium* genus [4] that can modulate the stress resilience of the holobiont [5].

The dark reactions of photosynthesis fix CO_2_ into organic carbon using the enzyme Ribulose-1,5-bisphosphate-carboxylase/oxygenase (RuBisCO). *Symbiodinium* contains a prokaryotic-type II RuBisCO, which has a low affinity for CO_2_ [6]–[9]. High concentrations of CO_2_ are therefore necessary to promote carbon assimilation and to meet the hosts' energetic demand for symbiont-derived photosynthates [10]–[12]. Holobiont respiration may present an additional internal CO_2_ source contributing to the complex carbon exchange and transfer system within corals. Chlororespiration, involving plastoquinone (PQ) oxidation with O_2_ and a terminal oxidase (PTOX) [13] can be active within the chloroplasts of *Symbiodinium*. Furthermore, calcification occurring in the calicodermis of the coral [14] and host mitochondrial respiration can further contribute to the internal CO_2_ supply in the holobiont [15], [16].

Coral host respiration is just one source of inorganic carbon for symbiont photosynthesis [17]–[19]; external inorganic carbon sources such as seawater are also utilised. However, the supply of inorganic carbon via passive diffusion from the surrounding seawater and host tissue is restricted by several factors: 1) the generally low CO_2_ content of seawater, 2) the presence of a diffusive boundary layer, and 3) the presence of multiple membranes of the host tissue surrounding the endodermal *Symbiodinium* cells, which need to be traversed. Both, coral host and symbionts employ a range of carbon concentrating mechanisms (CCMs) [20]–[24] to enhance the carbon supply from the external medium and thus increase CO_2_ availability to the *Symbiodinium* chloroplasts [25] as well as for calcification purposes [26].

The rate of photosynthesis by the symbionts and therefore their carbon demand is closely correlated with photon irradiance [27], and may become carbon limited under high irradiance [28]. As the delivery of carbon to the algal symbionts is controlled by the activity of CCMs (of coral host as well as algal symbionts), as well as host respiration [19], the host metabolism can thus have a strong impact on symbiont photosynthesis, e.g., by supplying sufficient inorganic carbon under high irradiance. While demands on the host-supplied carbon shift with irradiance, e.g., due to extra demand in light-enhanced calcification [29], there are only few experimental investigations of such responses in the literature [26], [30]. We investigated if respiratory-dependent processes in the coral would follow a typical asymptotic rise with increasing irradiance, as it is known for photosynthetic processes.

Photosynthesis and calcification require carbon as substrate [31], [32]; photosynthesis is directly dependent on light and coral calcification is known to be light-enhanced [33], [34]. Indeed, there is a close interplay of internal utilization of metabolically derived carbon for both processes. Carbonic anhydrase enzymes catalyse the reaction CO_2_+H_2_O ↔ HCO_3_^−^+H^+^, and therefore generate substrate for the calcification reaction (CO_2_+H_2_O+Ca^++^ ↔ CaCO_3_+2H^+^), as well as for photosynthesis: CO_2_+H_2_O ↔ CH_2_O+O_2_ [35], [36].

The exchange of respiratory gases (O_2_ and CO_2_) in photosynthetic symbioses is difficult to study in the light because respiratory O_2_ uptake is masked by the O_2_ production from photosynthesis. At low irradiance, where symbiont photosynthesis is lower than respiratory activity in the coral, i.e., below the irradiance compensation point net O_2_ uptake and CO_2_ release can be measured [37]. To measure these gas exchange patterns in corals is challenging, as several discrete ‘compartments’ of respiration operate in parallel and in close proximity, and therefore there is a close coupling between autotrophic and heterotrophic processes [38].

Enhanced post-illumination dark respiration (EPIR), which is the respiratory activity measured just after transition from light to darkness, has been used to support assumptions about light-driven respiratory processes in corals [16], [34]. However, in the absence of light there is no production of reducing agents due to the absence of photosynthetic light reactions, so that EPIR likely underestimates light respiration. To quantify respiration in the light, O_2_ microsensors can be used to quantify gross photosynthesis rates (GP_O2 micro_) in corals independent of respiration [14], [39], [40]. In conjunction with flux calculations of the net photosynthetic rate (Pnet_O2 micro_) from measured steady-state O_2_ concentration profiles, microsensor measurements allow for the determination of respiration rates in the light [41].

In this study, we present the first direct measurements of light respiration in corals as a function of irradiance. We combine O_2_ microsensor measurements with detailed CO_2_ exchange measurements to assess the relationship between CO_2_ exchange and symbiont gross photosynthesis rates in two scleractinian corals, *Pocillopora damicornis* (Linnaeus, 1758) and *Pavona decussata* (Dana, 1846), that are known to harbour the same *Symbiodinium* subclade (C1) [42]. The light dependency of external carbon uptake and respiratory activity was also examined, to see if respiratory processes followed an asymptotic rise with irradiance similar to photosynthetic processes.

## Materials and Methods

### Coral collection and preparation

Specimens of *Pocillopora damicornis* (Pocilloporidae) and *Pavona decussata* (Agariciidae) were collected from Heron Island reef flat (23° 26′ 60 S, 151° 55′ 0 E) (Great Barrier Reef Marine Park Authority collection permit G09/30854.1) and maintained for up to 2 months at the University of Technology Sydney. The coral *P. damicornis* is finely branched and highly sensitive to environmental factors that cause bleaching, while *P. decussata* is foliaceous (plate-like) and tolerant to environmental factors that cause bleaching [43]. After fragmentation of coral colonies, a number of similar sized pieces (average surface area: 28.6±11.3 cm^2^ and 23.5±7.2 cm^2^ for *P. damicornis* and *P. decussata*, respectively; mean ±s.e.m.; n = 3–4) were fixed with non-toxic epoxy (AquaKnead, Selleys, Australia) to sample holders. Corals were kept at 26±1°C under irradiance of ∼40 µmol photons m^−2^ s^−1^ (12 h: 12 h, light: dark cycle) in aquaria with recirculating artificial seawater (ASW; Aquasonic, Australia; salinity of 33 and a carbonate content of 140 ppm).

### Experimental setup

We used a novel instrumental array, a photobioreactor (PBR) (Gademann Instruments GmbH, Effeltrich, Germany), combining two metabolic gas exchange measuring techniques (O_2_ exchange and CO_2_ exchange). Only CO_2_ measurements are presented in this study. The setup consisted of a closed, continuously stirred thermostated chamber with a known volume of seawater containing a coral sample and an overlaying headspace [44]. The CO_2_ content in the overlying headspace of the chamber was measured on a calibrated infrared gas analyser (IRGA; MGA3000, ANRI instruments, Ferntree Gully, Victoria, Australia) with a 1 s sampling frequency. The sample chamber had a vertically mounted ‘warm white’ LED panel (NS2L123BT, Nichia, Japan) with 96 single-spot LEDs capable of applying up to 1500 µmol photons m^−2^ s^−1^ at the sample surface.

Dissolved CO_2_, as well as incident irradiance and temperature were measured for each specimen held in the PBR chamber. During PBR operation, the gas-phase effervesced through the liquid-phase to equilibrate dissolved CO_2_. CO_2_ concentration changes within the headspace of the PBR chamber were estimated according to Henry's gas law, which states that at a constant temperature and pressure the gas content between gas- and liquid-phase will move into a steady-state equilibrium. Measured CO_2_ concentrations (ppm) in the headspace were therefore used to calculate molar changes of CO_2_. The molar volume of CO_2_ (Vn) in the seawater was determined as follows: 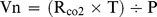
(1)where R_CO2_ is the specific CO_2_ gas constant 188.9 m^3^ Pa K^−1^ mol^−1^, T is the incubating temperature 26°C (299.15 K), and P is the ambient atmospheric pressure at sea level 1000 Pa [45]. In the measurement setup, Vn = 56.5 m^3^ mol^−1^. By dividing Vn with the molar mass of CO_2_ (44.01 g mol^−1^) the molar volume of CO_2_ per 1 ppm was then determined to be M = 1.3 mg m^3^. The measured CO_2_ concentrations in units of ppm could thus be converted to metric units and further into molar flux rates considering molar mass, the time of incubation, the volume of the gas-phase of the PBR, as well as the coral surface area. CO_2_ exchange was expressed as nmol CO_2_ cm^−2^s^−1^.

### Experimental protocol

At the beginning of the experiment, each coral specimen was incubated for ∼20 min in the PBR to account for the establishment of equilibrium between gas- and liquid- phase.

Photosynthesis–irradiance (P–E) curve measurements for *P. damicornis* (n = 4) and *P. decussata* (n = 3) began with a dark incubation to determine dark CO_2_ respiration rates followed by subsequent illumination using 9 photon irradiance levels (10, 20, 40, 78, 210, 360, 560, 780 and 1100 µmol-photons-m-2-s-1). Each illumination period lasted for 20 min and was followed by a 20 min dark incubation period. Incubation times were chosen to account for equilibration of gas- and liquid-phase. Gas exchange readings were taken from the last 5 min of each incubation interval.

Net CO_2_ uptake, measured during the light in the PBR, as well as respiratory CO_2_ production, measured during the dark in the PBR, were used to estimate gross CO_2_ exchange (G_CO2 PBR_). For an overview of parameters see Table 1.

**Table 1:** Overview of abbreviations and definition of gas exchange parameters from analyses with the photobioreactor (PBR) and from microsensor measurements.

Abbreviation	Parameter	Definition
GP_O2 micro_	*In hospite* gross O_2_ production	Measured using microsensor within the coral tissue as a direct measure
Pnet_O2 micro_	Net photosynthetic O_2_ production	Measured using microsensor above the coral tissue e.g. including O_2_ uptake processes
R_light O2 micro_	Light O_2_ respiration	Measured using microsensor measurements; determined through calculation of net and gross O_2_ production
R_dark O2 micro_	Steady-state O_2_ dark respiration	Measured using microsensor within the coral tissue as a direct measure after sufficient dark incubation; respiratory O_2_ consumption
G_CO2 PBR_	Gross CO_2_ exchange	Measured with the PBR; determined as the sum of net and respiratory CO_2_ exchange

### Oxygen microsensor measurements

We used O_2_ microsensors to quantify gross and net photosynthesis under a set of increasing photon irradiance levels. Corals were placed in an acrylic flow-through chamber (flow velocity ∼1 cm s^−1^) [46] with aerated, artificial seawater (see above). Samples were illuminated vertically using a fiber-optic tungsten-halogen lamp equipped with a heat filter and a collimating lens (KL-2500, Schott GmbH, Germany). The O_2_ microsensors were mounted on a PC-controlled motorized micromanipulator for automatic profiling (Pyro-Science GmbH, Germany) at an angle of 20° relative to the vertical incident light. A detailed description of the microsensor setup can be found in Wangpraseurt et al. [46]. Microscale O_2_ measurements were performed with Clark-type O_2_ microsensors (tip size: 25 µm; stirring sensitivity: <1%, 90% response time: <0.5 s; Unisense A/S, Aarhus, Denmark). Given that this high-precision technique requires more measuring time, only six photon irradiance levels (0, 40, 80, 210, 550, 1100 µmol photons m^−2^s^−1^) were applied for 20 min each (matching irradiance levels used in the PBR). At each irradiance, net and gross photosynthesis rates were determined by measuring steady-state O_2_ concentration profiles and O_2_ concentration dynamics under light-dark shifts, respectively [39], [40]. The O_2_ concentration profiles were measured from the coral surface upwards into the water column in vertical steps of 40 µm. Light-dark shifts were conducted from the coral surface down to the coral skeleton, which covered a distance of ∼80 µm for both species. The position of the sensor on the skeleton surface was identified as a slight bending of the microsensor. For each fragment, three locations at least 2 cm apart were randomly chosen and measurements were averaged. Measurements were exclusively conducted on the coenosarc (tissue connecting polyps) to minimize the influence of tissue movement [39].

Net O_2_ exchange fluxes were calculated from the measured steady-state concentration profiles using Fick's first law of diffusion with a molecular diffusion coefficient for O_2_ of 2.241×10^−5^ cm^2^ s^−1^ (25°C and salinity 33) [47]. Area-specific gross photosynthesis rates (GP_O2 micro_) were obtained by dividing the measurements of volume-specific GP with the thickness of the tissue, i.e. 80 µm (see above). The light respiration rate (R_light O2 micro_) was then calculated by subtracting the area-specific GP_O2 micro_ and net photosynthesis rate (Pnet_O2 micro_):
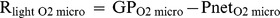
(2)


### Biometric measures

Following gas exchange measurements, coral specimens were snap frozen in liquid N_2_ for subsequent determinations of algal symbiont density, chlorophyll concentration and protein content. Once removed from the liquid N_2_, corals were transferred to a 100 mL Erlenmeyer flask and kept on ice, with 15 mL of homogenization buffer (4°C) consisting 1 mM phenylmethylsulfonyl fluoride (protease inhibitor) in 0.2 µm-filtered seawater (FSW). The flask was sealed with Parafilm and shaken for 10 min by hand in a circular motion, allowing the coral tissue to be torn off the skeleton. The resulting liquid was homogenized on ice (Ultra-Turrax, Ika, Rawang, Malaysia) for 30 s. The homogenate was centrifuged at 700 g for 5 min at 4°C and the resulting pellet of *Symbiodinium* was retained for algal cell density counts and for chlorophyll concentration analyses (see below). The supernatant contained coral tissue remains, of which 2 mL were sampled for protein content determination using the Bradford assay, with bovine serum albumin standards [48]. Protein assay absorbance was measured at 595 nm with a 96-well plate reader (Bio Rad Bench Mark Plus spectrophotometer, Hercules, California, USA) and analysed using the Microplate Manager Software (Bio Rad, Hercules, California, USA).

The *Symbiodinium* pellet was re-suspended in 4 mL of FSW and subsamples were taken for algal symbiont counts according to Edmunds and Gates [49]. The algal suspension was again centrifuged at 1789 g and the pellet re-suspended in 3 mL of 90% acetone and incubated for 24 h at 4°C to extract pigments. Chlorophyll *a* and *c*_2_ concentrations were measured using a spectrophotometer (Cary UV-VIS, Agilent Technologies, Australia) using absorbance readings according to Ritchie [50]. The coral skeleton surface area was determined using the single-dip paraffin wax technique [51].

### Statistical analyses

Differences in biometric parameters between the two coral species were analysed using Student's t-test (t; α = 0.05). Differences in respiratory rates were determined by using univariate one-way and two-way analysis of variance (ANOVA; F; α = 0.05). ANOVA assumptions for normal distribution and homogeneity of variance were tested using Shapiro Wilk and Levene's tests, respectively. Tukey's honest significant difference (HSD) test (t; α = 0.05) was used for post-hoc comparison of means to identify differences at 95% confidence interval. All statistical analyses were carried out using Statistica 10 (Statsoft Inc., Tulsa, OK, USA).

## Results

### Biometric measures

The two coral species differed in protein content, total chlorophyll (Chl; Chl *a*+*c*_2_) concentration, as well as algal symbiont cell density (Table 2). The coral *P. decussata* displayed a significantly higher protein concentration than *P. damicornis* (t(6)  = 3.925, p = 0.026). Further, *P. decussata* contained more than twice the total Chl concentration (mg cm^−2^) (t(6)  = 3.83, p = 0.009) and harboured significantly higher algal cell densities than *P. damicornis* (t(6)  = 2.73, p = 0.034). However, *Symbiodinium* in both species contained similar amounts of total Chl cell^−1^.

**Table 2:** Biometric measures of the hard corals *Pocillopora damicornis* and *Pavona decussata,* displaying total protein content (mg cm^−2^), total chlorophyll per area (Chl *a*+*c*_2_) (mg cm^−2^), algal cell densities (cells cm^−2^) and total Chl per cell (pg cell^−1^) (n = 4; mean ±s.e.m.).

	*Pocillopora damicornis*	*Pavona decussate*
**Total protein (mg cm^-2^)**	1.19±0.22	2.31±0.36*
**Chl (mg cm^−2^)**	0.003±0.001	0.007±0.001*
**Algal cell densities (cells cm^−2^)**	5.32×10^5^±1.91×10^5^	16.7×10^5^±2.00×10^5^*
**Total Chl (pg cell^−1^)**	9.735±1.509	9.666±2.463

* Significantly different values are indicated with an asterisk.

### Gross CO_2_ exchange

**Figure 1:**
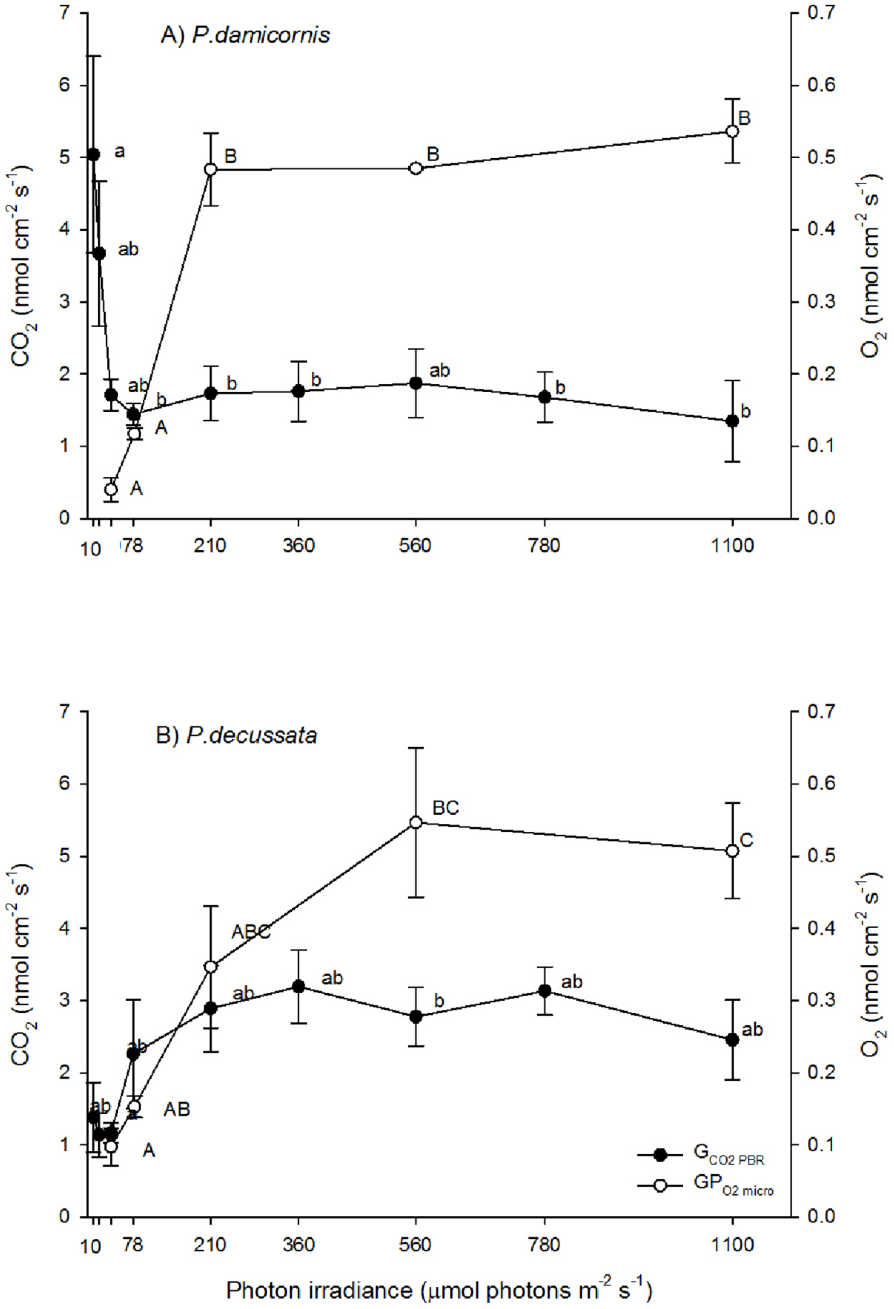
Variation in gas exchange measurements with irradiance. The graphs display gross CO_2_ exchange (G_CO2 PBR_; black circles; CO_2_ nmol cm^−2^ s^−1^) and microsensor derived gross photosynthetic O_2_ production (GP_O2 micro_; open circles; O_2_ nmol cm^−2^ s^−1^) of the hard coral species *Pocillopora damicornis* (A) and *Pavona decussata* (B) as a function of nine irradiances (mean ±s.e.m.; G_CO2 PBR_: n = 4 and GP_O2 micro_: n = 2); Tukey honest significant difference test results are indicated for G_CO2 PBR_ (lower case letters) and GP_O2 micro_ (capitals) (p<0.05).

For *P. damicornis*, gross CO_2_ uptake from the seawater declined up to an irradiance of 78 µmol photons m^−2^ s^−1^ (one-way ANOVA, F (8, 27)  = 2.90, p = 0.018; Fig. 1 A), and then increased slightly up to an irradiance of 560 µmol photons m^−2^ s^−1^ followed by a small but significant decline at irradiances of 780 and 1100 µmol photons m^−2^ s^−1^ (Tukey HSD, p<0.05; Fig. 1 A, also see Table S1). In contrast, gross CO_2_ uptake of *P. decussata* showed no decline in the first phase of illumination (10 and 20 µmol photons m^−2^ s^−1^). Gross CO_2_ uptake increased from an irradiance of 40 up to 78 µmol photons m^−2^ s^−1^ and then remained steady besides a dip at an irradiance of 560 µmol photons m^−2^ s^−1^ (Fig. 1 B). The metabolic activity differed most significantly between the two species under low irradiance (pooled CO_2_ rates for irradiances of 10–40 µmol photons m^−2^ s^−1^; t (22)  = 3.54, p<0.001; Figs. 1 A and B, also see Table S1).

Microsensor measurements of gross photosynthesis, GP_O2 micro_ in *P. damicornis* revealed maximum rates of 0.502±0.017 nmol O_2_ cm^−2^ s^−1^ at irradiances of 210 µmol photons m^−2^ s^−1^ (one-way ANOVA, F(4,5)  = 115.06, p<0.001; Tukey HSD; p<0.05; Fig. 1 A, also see Table S1). In *P. decussata*, GP_O2 micro_ increased more gradually reaching a maximum at an irradiance of 560 µmol photons m^−2^ s^−1^ (one-way ANOVA, F(4,5)  = 8.9182, p = 0.017; Tukey HSD, p<0.05) with an average GP_O2 micro_ rate of 0.527±0.020 nmol O_2_ cm^−2^ s^−1^ (Fig. 1 B). In both species we did not detect down-regulation of GP_O2 micro_ at above saturating irradiance levels (i.e. up to 1100 µmol photons m^−2^ s^−1^).

### Respiration

**Figure 2:**
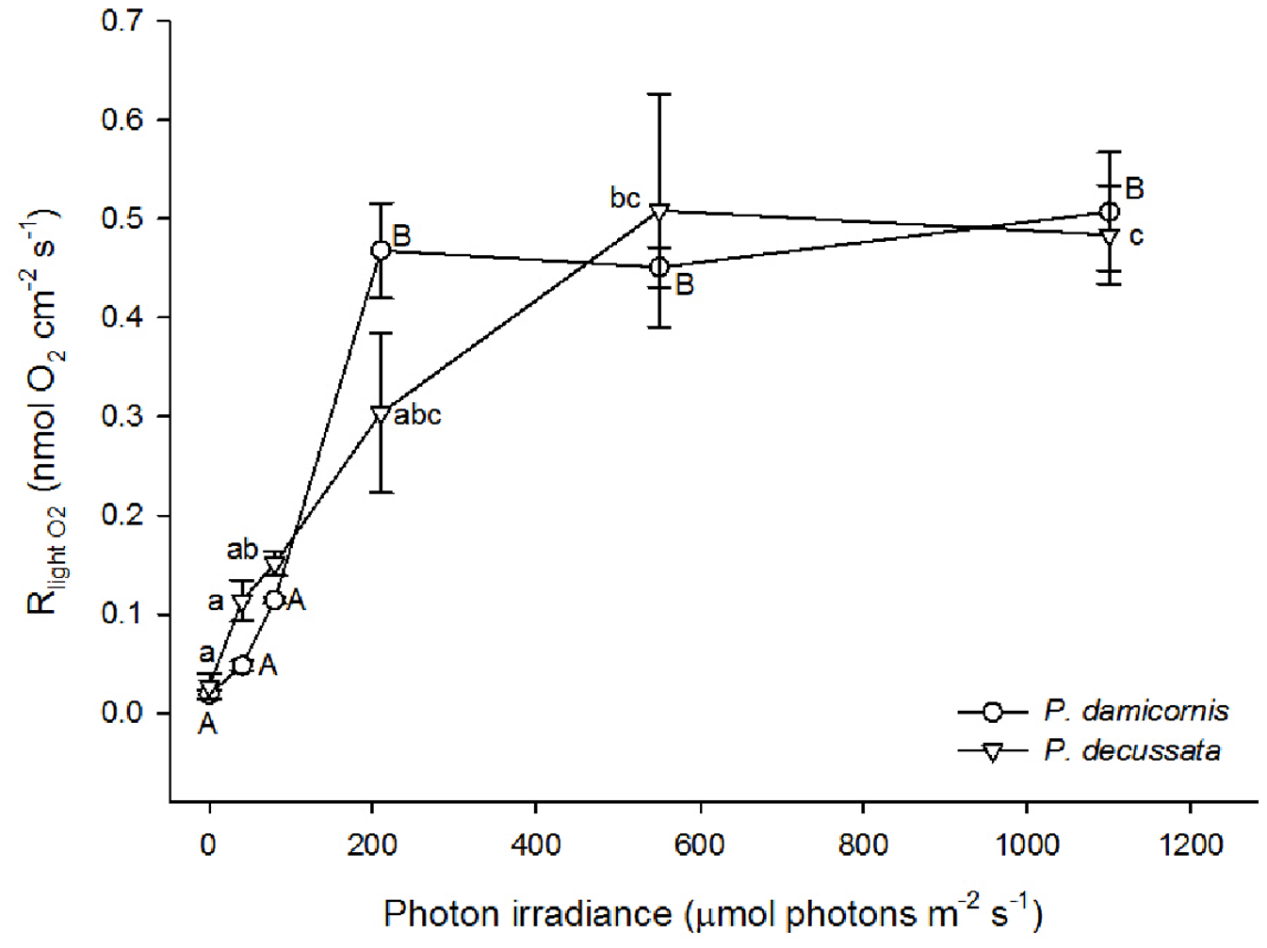
Light respiration (R_light O2 micro_) of the hard coral species *Pocillopora damicornis* (clear circle) and *Pavona decussata* (clear triangle) are displayed as a function of 6 irradiances (mean ±s.e.m.; n = 2). Tukey honest significance difference test results are indicated, where capital letters are describing groupings for *P. damicornis* and lower case letters groupings of *P. decussata* (p<0.05).

Light respiration (R_light O2 micro_) increased with increasing irradiance (Fig. 2), with a maximum R_light O2 micro_ of ∼0.5 nmol O_2_ cm^−2^ s^−1^ for both coral species (one-way ANOVA, F(5,6)  = 10.26; p = 0.007 for *P. decussata* and F(5)  = 101.08; p<0.001 for *P. damicornis*). However, R_light O2 micro_ increased more rapidly with irradiance in *P. damicornis* than in *P. decussata* (Fig. 2, also see Table S1).

A comparison of R_light O2 micro_ with R_dark O2 micro_ revealed a strong light response at photon irradiances >210 µmol photons m^−2^ s^−1^ in both species (data not displayed). Where the increase in light-driven respiration rates compared to dark respiration rates was greater in *P. damicornis* than it was found for *P. decussata*. For example, at 210 µmol photons m^−2^ s^−1^ light respiration increased 25 times in *P. damicornis* but only 11 times in *P. decussata*.

The ratio of R_light O2 micro_ to microsensor derived gross photosynthesis (GP_O2 micro_) differed between the two species. The maximum R_light O2 micro_ constituted ∼97% of GP_O2 micro_ in *P. damicornis*, while it only accounted for ∼88% in *P. decussata.*

## Discussion

**Figure 3:**
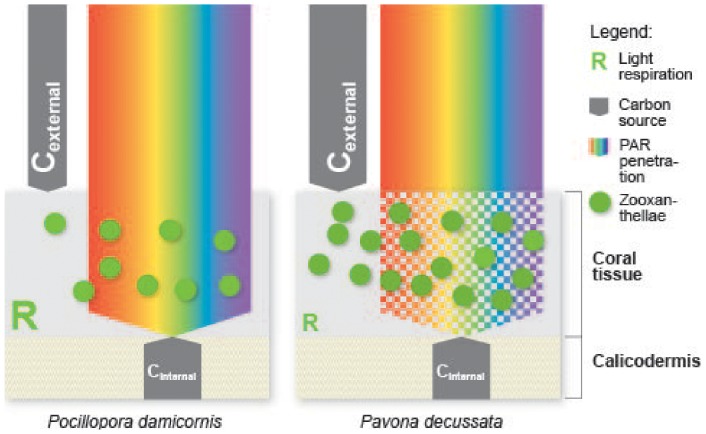
Conceptual model of light and carbon availability, in the two hard coral species, *Pocillopora damicornis* and *Pavona decussata* in moderate light (∼100 µmol photons m^−2^ s^−1^). The schematic diagram of a coral shows the coral tissue containing algal symbionts (green circles), which lies above the calicoblastic layer. Photosynthetic active radiation (PAR) (rainbow arrow) penetrates the coral tissue. In *P. decussata* a higher density of symbionts reduced light availability compared to *P. damicornis*. Dissolved inorganic carbon (grey arrows; quantity is relative to arrow thickness) can originate from internal sources such as the calicoblastic layer or from the external environment, where *P. decussata* draws stronger on the external carbon uptake. Light respiration (R) (strength indicated through size), was greater in *P. damicornis* than in *P. decussata*.

This is the first study reporting an integrated approach measuring coral light respiration and gross photosynthesis with O_2_ microsensors and CO_2_ gas exchange techniques across a range of irradiance. The two main finding of this study are that i) light-saturated (at 210 µmol photons m^−2^ s^−1^) respiration rates (R_light O2 micro_) were multiple times higher than steady-state dark respiration rates (R_dark O2 micro_) (11 times for *P. decussata* and 25 times for *P. damicornis*, and ii) *P. damicornis* and *P. decussata* differ in their photophysiological function despite likely harbouring the same symbiont subclade C1 [42] (see Fig. 3 for a conceptual diagram of the main findings).

Sufficient supply of CO_2_ to the algal symbionts is of paramount importance for the functioning of a coral symbiosis [18], [52], [53], where an increased supply enhances photosynthesis [31]. Gross photosynthesis rates (GP_O2 micro_) were similar for both coral species across the applied irradiance levels. However, gross CO_2_ uptake rates, as well as algal symbiont density were generally higher in *P. decussata* (Fig. 1 B). These results raise the question as to why a coral with twice as many symbionts and greater CO_2_ uptake (*P. decussata*) did not show a greater photosynthetic productivity. The coral *P. decussata* had a much greater protein biomass than the coral *P. damicornis* and the algal symbionts would have been more densely packed within the coral tissue. Self-shading of the algal symbionts [54], as well as species-specific differences in light propagation within the host tissue [46], [55] could explain our findings for *P. decussata*. A model of how canopy-understory development can influence the photosynthesis-irradiance (P-I) relationship has previously been introduced [56]. Here we could expand that model to introduce the light respiratory activity as well as carbon uptake in relation to how canopy-understory influences the P-I relationship in the two corals examined here (see Fig. 3).

Light respiration in *P. damicornis* reached its maximum at a lower irradiance than in *P. decussata* and exceeded dark respiration (Fig. 2). A higher proportion of GP_O2 micro_ was therefore contributed by light respiration in *P. damicornis* than in *P. decussata*. Our results suggest therefore that species-specific light-driven respiratory processes are active within the two coral species.

Light-driven respiration is often coupled to calcification in the calicodermis [14], [29], [33], [36], [57] and it seems possible that the calcification process accounts for a large fraction of the light respiration. For calcification to take place, O_2_ and photosynthate are necessary so that the coral host can liberate adenosine-triphospate (ATP) for the calcifying process [58], [59]. The hyperbolic increase in light respiration for both species, up to the maximum measured photon irradiance (1100 µmol photons m^−2^ s^−1^; Fig. 2) suggests that host respiration is closely coupled to release of photosynthates from zooxanthellae. However, recent attempts to investigate calcification and light respiration rates in corals, using an indirect measuring technique, found that light respiration increased the most in zooxanthellae as opposed to the coral host [60]. Given these results, it seems more likely that metabolic activity supporting calcification, e.g., *Symbiodinium*'s photosynthetic reaction and carbon fixation, are responsible for most of the increase in light respiration. Calcification itself is a positive feedback mechanism for *Symbiodinium* photosynthesis, as CO_2_ is being produced during skeleton accretion [29]. Both species showed steady and light-independent gross CO_2_ uptake rates at >78 µmol photons m^−2^ s^−1^, where calcification could then fuel the photosynthetic activity through internal carbon release. However, the recently proposed ‘proton flux hypothesis’ [36], where the shedding of protons generated during the calcification process is proposed to result in a lag of CO_2_ uptake could also explain our results. Whether light respiration is simply controlled by the availability and source of carbon substrates or other metabolic controls remains to be investigated.

In both corals, *P. damicornis* and *P. decussata*, light-saturated respiration rates (R_light O2 micro_) at 210 µmol photons m^−2^ s^−1^ were similar. Light stimulated respiration in *P. damicornis* increased to a greater degree than that in *P. decussata* (25 versus 11 times). Light-saturated respiration rates in both species reached an asymptotic value of 5 nmol cm^−2 ^s^−1^ at photon irradiances >210 µmol photons m^−2^ s^−1^ (Fig. 2). The strong increase of respiration rates during the light as compared to steady-state dark respiration rates are most likely due to the low-light acclimation of the experimental corals (40 µmol photons m^−2^ s^−1^). Dark respiration rates are generally dependent upon pre-experimental incubation irradiances [61], [62]. Under low light adaptation steady-state dark respiration rates are low but once exposed to light, the metabolic activity increases and so do light respiration rates and other oxygen uptake processes. The magnitude of this increase is independent on the pre-experimental incubation irradiance [62].

Photoacclimation is a process of morphological (here in terms of coral host) and physiological adjustments of a phototrophic organism towards growth irradiances. Pigmentation (coral host pigmentation [63] and light harvesting pigments such as accessory pigments and chlorophyll [64]), as well as photochemical quenching capacity (xanthophyll pool [65], [66]) can be increased and decreased in abundance and concentrations. During high light exposure these adjustments help acclimatization in the phototroph only to some extend, and as a result, high light stress results in the accumulation of reactive oxygen species [67], the stimulation of alternative electron transport systems [68], [69], often consuming oxygen, and of photorepair mechanisms [70], [71]. The cost of all these processes results in low net photosynthesis [62], due to increased respiration and other oxygen uptake [39], [72]. The light source in the experiments of this study excluded the naturally occurring ultraviolet radiation, which corals experience in the field and which is a major cause of photodamage [73], [74]. Translating our findings to corals in the field, the increase of oxygen uptake rates on going from dark to light (or from low to high light) might therefore not be as great as found in this study; however, once photorepair processes are entrained the actual oxygen uptake rates might be just as high or even higher.

Pronounced stimulation of respiration in light has been reported for the coral species *Galaxea fascicularis*, where light respiration was ∼12 times higher than dark respiration under an irradiance of 140 µmol photons m^−2^ s^−1^ [14]. Kühl et al. [39] observed values of light respiration to be ∼6 times higher than during dark respiration in *Favia* sp. under an irradiance of 350 µmol photons m^−2^ s^−1^. Here light respiration accounted for 77% of the gross photosynthetic O_2_ production. The differing increase of respiration rates from dark to light between the reporting studies and our results are probably due to species differences and differential pre-experimental and experimental irradiances. In our study light respiration accounted for 88% of gross photosynthetic O_2_ production in *P. decussata* and 97% of gross photosynthetic O_2_ production in *P. damicornis* at 210 µmol photons m^−2^ s^−1^. Maximum gross photosynthetic O_2_ production were on average ∼0.53 nmol O_2_ cm^−2^ s^−1^ for both coral species (Fig. 1) and were of a similar magnitude to other microsensor measurements of gross photosynthesis rates in corals [75].

Light dependent increase in O_2_ consumption through respiratory processes has been discussed previously [68]. Tchernov et al. [68] concluded that ongoing activity of the MAP cycle could be accounted for by the increased O_2_ uptake with increasing photon irradiance. Indeed, various light-driven O_2_ consuming processes, such as photorespiration [76], [77] and the MAP cycle [68], [78], [79] could also be involved in the high level of light respiration observed here. However, the activity of the MAP cycle does not result in net O_2_ concentration changes [78]; it therefore cannot be measured in O_2_ exchange measurements with microsensors [80]. Hence, we conclude that the only other process to explain the light respiration results apart from light-stimulated mitochondrial O_2_ uptake is photorespiration, involving oxygenase activity of RuBisCO [81]. However, further investigations are needed to verify and describe these processes.

## Conclusions

Light-saturated respiration rates (R_light O2 micro_) were similar in both corals and multiple times higher than steady-state dark respiration rates (R_dark O2 micro_). This is interpreted as the activity of light-driven metabolic pathways that increase with increasing irradiance. The light respiration rates show, that differential CO_2_ uptake rates of the two species examined could indicate that carbon availability influences the metabolic processes of the holobiont. Although both coral hosts are known to harbour the same *Symbiodinium* subclade C1 [42], it seems that they experience different host-specific microenvironmental conditions (see Figure 3).

## Supporting Information

Table S1 **Gas exchange rates measured as a function of irradiance for** ***Pocillopora damicornis*** **and** ***Pavona decussata*** **.** Following gas exchange rates are presented: GP_O2 micro_ – *In hospite* gross O_2_ production (microsensor based), Pnet_O2 micro_ – net photosynthetic O_2_ production (microsensor based), R_light O2 micro_ – light O_2_ respiration (microsensor based), G_CO2 PBR_ – Gross CO_2_ exchange for 6 light intensities.
(DOCX)

## References

[1] Muscatine L (1990) The role of symbiotic algae in carbon and energy flux in reef corals. In: Dubinsky Z, editor.Ecosystems of the World: Coral reefs.Amsterdam: Elsevier. pp. 75–87.

[2] Muscatine L, Porter JL (1977) Reef corals: mutualistic symbioses adapted to nutrient-poor environments. BioScience 27: 454–460.

[3] Pernice M, Meibom A, Van Den Heuvel A, Kopp C, Domart-Coulon I, et al (2012) A single-cell view of ammonium assimilation in coral–dinoflagellate symbiosis. International Society for Microbial Ecology 6: 1314–1324. 10.1038/ismej.2011.196 PMC3379633 22222466

[4] Sampayo EM, Dove S, LaJeunesse TC (2009) Cohesive molecular genetic data delineate species diversity in the dinoflagellate genus *Symbiodinium* . Molecular Ecology 18: 500–519. 19161470 10.1111/j.1365-294X.2008.04037.x

[5] Fisher P, Malme MK, Dove S (2012) The effect of temperature stress on coral - *Symbiodinium* associations containing distinct symbiont types. Coral Reefs 31: 473–485.

[6] Yellowlees D, Rees TA, Leggat W (2008) Metabolic interactions between algal symbionts and invertebrate hosts. Plant, Cell & Environment 31: 679–694. 10.1111/j.1365-3040.2008.01802.x 18315536

[7] Whitney SM, Yellowlees D (1995) Preliminary investigations into the structure and activity of ribulose biphosphate carboxylase from two photosynthetic dinoflagellates. Journal of Phycology 31: 128–146.

[8] Rowan R, Whitney SM, Fowler A, Yellowlees D (1996) Rubisco in marine symbiotic dinoflagellates: form II enzymes in eukaryotic oxygenic phototrophs encoded by a nuclear multigene family. The Plant Cell 8: 539–553. 8721755 10.1105/tpc.8.3.539 PMC161119

[9] Raven JA, Beardall J (2003) Carbon acquisition mechanisms in algae: carbon dioxide diffusion and carbon dioxide concentrating mechanisms. In: Larkum AWD, Douglas AE, Raven JA, editors.Photosynthesis in Algae.Dordrecht, The Netherlands: Kluwer. pp. 225–244.

[10] Raven JA (2003) Inorganic carbon concentrating mechanisms in relation to the biology of algae. Photosynthesis Research 77: 155–171. 16228373 10.1023/A:1025877902752

[11] Lilley RM, Ralph PJ, Larkum AWD (2010) The determination of activity of the enzyme Rubisco in cell extracts of the dinoflagellate alga *Symbiodinium* sp. by manganese chemiluminescence and its response to short-term stress of the alga. Plant, Cell & Environment 33: 995–1004. 10.1111/j.1365-3040.2010.02121.x 20102538

[12] Leggat W, Marendy EM, Baillie B, Whitney SM, Ludwig M, et al (2002) Dinoflagellate symbioses: strategies and adaptations for the acquisition and fixation of inorganic carbon. Functional Plant Biology 29: 309–322. 10.1071/PP01202 32689478

[13] Peltier G, Cournac L (2002) Chlororespiration. Annual Review of Plant Physiology Biology 53: 523–550. 10.1146/annurev.arplant.53.100301.135242 12227339

[14] Al-Horani FA, Al-Moghrabi SM, de Beer D (2003) The mechanism of calcification and its relation to photosynthesis and respiration in the scleractinian coral *Galaxea fascicularis* . Marine Biology 142: 419–426.

[15] Anthony KRN, Hoegh-Guldberg O (2003) Kinetics of photoacclimation in corals. Oecologia 134: 23–31. 12647175 10.1007/s00442-002-1095-1

[16] Cooper TF, Ulstrup KE, Dandan SS, Heyward AJ, Kühl M, et al (2011) Niche specialization of reef-building corals in the mesophotic zone: metabolic trade-offs between divergent *Symbiodinium* types. Proceedings of the Royal Scoiety B: Biological Sciences 278: 1840–1850. 10.1098/rspb.2010.2321 PMC3097838 21106586

[17] Muller-Parker G, D'Elia CF (1997) Interactions between corals and their symbiotic algae. In: Birkeland C, editor. Life and Death of Coral Reefs. New York: Chapman and Hall. pp. 96–113.

[18] Muscatine L, Porter JW, Kaplan IR (1989) Resource partitioning by reef corals as determined from stable isotope composition: I. delta ^13^C of zooxanthellae and animal tissue vs. depth. Marine Biology 100: 185–193.

[19] Yellowlees D, Warner M (2003) Photosynthesis in Symbiotic Algae. In: Larkum AWD, Douglas SE, Raven JA, editors.Photosynthesis in Algae. Dordrecht, Netherlands: Kluwer Academic Publishers.

[20] Al-Moghabri S, Goiran C, Allemand D, Speziale N, Jaubert J (1996) Inorganic carbon uptake for photosynthesis by the symbiotic coral-dinoflagellate association II. mechanisms for bicarbonate uptake. Journal of Experimental Marine Biology and Ecology 199: 227–248.

[21] Rands ML, Loughman BC, Douglas AE (1993) The symbiotic interface in an alga-invertebrate symbiosis. Proceedings of the Royal Scoiety B: Biological Sciences 253: 161–165.

[22] Leggat W, Badger MR, Yellowlees D (1999) Evidence for an inorganic carbon-concentrating mechanism in the symbiotic dinoflagellate *Symbiodinium* sp. Plant Physiology 121: 1247–1255. 10594111 10.1104/pp.121.4.1247 PMC59491

[23] Bertucci A, Tambutté E, Supuran CT, Allemand D, Zoccola D (2011) A new coral carbonic anhydrase in *Stylophora pistillata* . Marine Biotechnology 13: 992–1002. 21318259 10.1007/s10126-011-9363-x

[24] Graham D, Smillie RM (1976) Carbonate dehydratase in marine organisms of the Great Barrier Reef. Australian Journal of Plant Physiology 3: 153–179.

[25] Moya A, Tambutte S, Bertucci A, Tambutte E, Lotto S, et al (2008) Carbonic anhydrase in the scleractinian coral *Stylophora pistillata*- characerization, localization, and role in biomineralization. Journal of Biological Chemistry 283: 25475–25484. 18617510 10.1074/jbc.M804726200

[26] Furla P, Galgani I, Durand I, Allemand D (2000) Sources and mechanisms of inorganic carbon transport for coral calcification and photosynthesis. The Journal of Experimental Biology 203: 3445–3457. 11044383 10.1242/jeb.203.22.3445

[27] Spencer Davies P (1991) Effect of daylight variations on the energy budgets of shallow-water corals. Marine Biology 108: 137–144.

[28] Reynaud S, Ferrier-Pages C, Sambrotto R, Juillet-Leclerc A, Jaubert J, et al (2002) Effect of feeding on the carbon oxygen isotopic composition in the tissues and skeleton of the zooxanthellate coral *Stylophora pistillata* . Marine Ecology Progress Series 238: 81–89.

[29] Gattuso J-P, Allemand D, Frankignoulle M (1999) Photosynthesis and calification at cellular, organismal and community levels in coral reefs: a review on interactions and control by carbonate chemistry. American Zoologist 39: 160–183.

[30] Weis VM, Smith GJ, Muscatine L (1989) A “CO_2_ supply” mechanism in zooxanthellate cnidarians: role of carbonic anhydrase. Marine Biology 100: 195–202.

[31] Herfort L, Thake B, Taubner I (2008) Bicarbonate stimulation of calcification and photosynthesis in two hermatypic corals. Journal of Phycology 44: 91–98. 27041045 10.1111/j.1529-8817.2007.00445.x

[32] Buxton L, Badger MR, Ralph PJ (2009) Effects of moderate heat stress and dissolved inorganic carbon concentration on photosynthesis and respiration of *Symbiodinium* sp. (Dinophyceae) in culture and in symbiosis. Journal of Phycology 45: 357–365. 27033814 10.1111/j.1529-8817.2009.00659.x

[33] Moya A, Tambutte S, Tambutte E, Zoccola D, Caminiti N, et al (2006) Study of calcification during a daily cycle of the coral *Stylophora pistillata*: implications for ‘light-enhanced calcification’. The Journal of Experimental Biology 209: 3413–3419. 16916976 10.1242/jeb.02382

[34] Levy O, Dubinsky Z, Schneider K, Achituv Y, Zakai D, et al (2004) Diurnal hysteresis in coral photosynthesis. Marine Ecology Progress Series 268: 105–117.

[35] Jokiel PL (2011) The reef coral two compartment proton flux model: A new approach relating tissue-level physiological processes to gross corallum morphology. Journal of Experimental Marine Biology and Ecology 409: 1–12.

[36] Jokiel PL, Jury CP, Rodgers KS (2014) Coral-algae metabolism and diurnal changes in the CO_2_- carbonate system of bulk sea water. Peer J 2: e378. 24883243 10.7717/peerj.378 PMC4034600

[37] Sverdrup HU (1953) On conditions of vernal blooming of phytoplankton. Journal du Conseil/Conseil Permanent International pour l'Exploration de la Mer 18: 287–295.

[38] Tremblay P, Grover R, Maguer JF, Hoogenboom M, Ferrier-Pages C (2014) Carbon translocation from symbiont to host depends on irradiance and food availability in the tropical coral *Stylophora pistillata* . Coral Reefs 33: 1–13.

[39] Kühl M, Cohen Y, Dalsgaard T, Jørgensen BB, Revsbech NP (1995) Microenvironment and photosynthesis of zooxanthellae in scleractinian corals studies with microsensors for O_2_, pH and light. Marine Ecology Progress Series 117: 159–172.

[40] Revsbech NP, Jørgensen BB (1983) Photosynthesis of benthic microflora measured with high spatial resolution by the oxygen microprofile method: capabilities and limitations of the method. Limnology and Oceanography 28: 749–756.

[41] Jensen J, Revsbech NP (1989) Photosynthesis and respiration of a diatom biofilm cultured in a new gradient growth chamber. FEMS Microbiology Ecology 62: 29–38.

[42] Hill R, Ulstrup KE, Ralph PJ (2009) Temperature induced changes in thylakoid membrane thermostability of cultured, freshly isolated, and expelled zooxanthellae from scleractinian corals. Bulletin of Marine Science 85: 223–244.

[43] Hill R, Larkum AWD, Prášil O, Kramer DM, Kumar V, et al (2012) Light-induced redistribution of antenna complexes in the symbionts of scleractinian corals correlates with sensitivity to coral bleaching. Coral Reefs 31: 963–975.

[44] Schrameyer V (2013) Defining the bio-energetic limits of Symbiodinium sp.'s host-symbiont relationship under future climate scenarios [Doctor of Philosophy]. Sydney: University of Technology, 320 p. http://hdl.handle.net/10453/24110.

[45] Godish T (2004) Air Quality: Lewis Publisher CRC Press.

[46] Wangpraseurt D, Larkum AWD, Ralph PJ, Kühl M (2012) Light gradients and optical microniches in coral tissues. Frontiers in Microbiology 3: 316. 22969755 10.3389/fmicb.2012.00316 PMC3427877

[47] Li YH, Gregory S (1974) Diffusion of ions in sea water and in deep-sea sediments. Geochimica Cosmochim Acta 38: 703–714.

[48] Bradford MM (1976) A rapid and sensitive method for the quantitation of microgram quantities of protein utilizing the principle of protein-dye binding. Analytical Biochemistry 72: 248–254. 942051 10.1016/0003-2697(76)90527-3

[49] Edmunds PJ, Gates RD (2002) Normalizing physiological data for scleractinian corals. Coral Reefs 21: 193–197.

[50] Ritchie RJ (2006) Consistent sets of spectrophotometric chlorophyll equations for acetone, methanol and ethanol solvents. Photosynthesis Research 89: 27–41. 16763878 10.1007/s11120-006-9065-9

[51] Stimson J, Kinzie RA (1991) The temporal pattern and rate of release of zooxanthellae from the reef coral *Pocillopora damicornis* (Linnaeus) under nitrogen-enrichment and control conditions. Journal of Experimental Biology 153: 66–74.

[52] Weis VM (1993) Effect of dissolved inorganic carbon concentration on the photosynthesis of the symbiotic sea anemone *Aiptasia pulchella* Carlgren: role of carbonic anhydrase. Journal of Experimental Marine Biology and Ecology 174: 209–225.

[53] Goiran C, Al-Moghabri S, Allemand D, Jaubert J (1996) Inorganic carbon uptake for photosynthesis by the symbiotic coral/dinoflagellate association I. photosynthetic performances of symbionts and dependence on sea water bicarbonate. Journal of Experimental Marine Biology and Ecology 199: 207–225.

[54] Enriquez S, Mendez ER, Iglesias-Prieto R (2005) Multiple scattering on coral skeletons enhances light absorption by symbiotic algae. Limnology and Oceanography 50: 1025–1032.

[55] Wangpraseurt D, Larkum AWD, Franklin J, Szabo M, Ralph PJ, et al (2013) Lateral light transfer ensures efficient resources distribution in symbiont-bearing corals. Journal of Experimental Biology 217: 489–498. 10.1242/jeb.091116 24523498

[56] Jokiel PL, Morrissey JL (1986) Influence of size on primary production in the reef coral *Pocillopora damicornis* and the tropical macroalga *Acanthophora spicifera* . Marine Biology 91: 15–26.

[57] Marubini F, Barnett H, Langdon C, Atkinson MJ (2001) Dependence of calcification on light and carbonate ion concentration for the hermatypic coral *Porites compressa* . Marine Ecology Progress Series 220: 153–162.

[58] Allemand D, Ferrier-Pagès C, Furla P, Houlbrèque F, Puverel S, et al (2004) Biomineralisation in reef-building corals: from molecular mechanisms to environmental control. General Palaeontology (Palaeobiochemistry) 3: 453–467.

[59] Holcomb M, Tambutte E, Allemand D, Tambutte S (2014) Light enhanced calcification in *Stylophora pistillata*: effects of glucose, glycerol and oxygen. PeerJ 2: e375. 24883242 10.7717/peerj.375 PMC4034610

[60] Agostini S, Fujimura H, Fujita K, Suzuki Y, Nakano Y (2013) Respiratory electron transport system activity in symbiotic corals and its link to calcification. Aquatic Biology 18: 125–139.

[61] Anthony KRN, Hoegh-Guldber O (2003) Variation in coral photosynthesis, respiration and growth characteristics in contrasting light microhabitats: an analogue to plants in forest gaps and understoreys? Functional Ecology 17: 246–259.

[62] Hoogenboom MO, Anthony KRN, Connolly SR (2006) Energetic cost of photoinhibition in corals. Marine Ecology Progress Series 313: 1–12.

[63] Salih A, Larkum AWD, Cox G, Kühl M, Hoegh-Guldberg O (2000) Fluorescent pigments in corals are photoprotective. Nature 408: 850–853. 11130722 10.1038/35048564

[64] Krämer WE, Caamaño-Ricken I, Richter C, Bischof K (2012) Dynamic regulation of photoprotection determines thermal tolerance of two phylotypes of *Symiodinium* clade A at two photon fluence rates. Photochemistry and Photobiology 88: 398–413. 22117932 10.1111/j.1751-1097.2011.01048.x

[65] Brown BE, Ambarsari I, Warner ME, Fitt WK, Dunne RP, et al (1999) Diurnal changes in photochemical efficiency and xanthophyll concentrations in shallow water reef corals: evidence for photoinhibition and photoprotection. Coral Reefs 18: 99–105.

[66] Warner ME, Chilcoat GC, McFarland FK, Fitt WK (2002) Seasonal fluctuations in the photosynthetic capacity of photosystem II in symbiotic dinoflagellates in the caribbean reef-building coral *Montastrea* . Marine Biology 141: 31–38.

[67] Lesser MP (1997) Oxidative stress causes coral bleaching during exposure to elevated temperatures. Coral Reefs 16: 187–192.

[68] Tchernov D, Gorbunov MY, de Vagas C, Yadav SN, Milligan AJ, et al (2004) Membrane lipids of symbiotic algae are diagnostic of sensitivity to thermal bleaching in corals. Proceedings of the National Academy of Sciences 101: 13531–13535. 10.1073/pnas.0402907101 PMC518791 15340154

[69] Levy O, Achituv Y, Yacobi YZ, Dubinsky Z, Stambler N (2006) Diel ‘tuning’ of coral metabolism: physiological responses to light cues. Journal of Experimental Biology 209: 273–283. 16391349 10.1242/jeb.01983

[70] Hill R, Brown cM, DeZeeuw K, Campbell dA, Ralph PJ (2011) Increased rate of D1 repair in coral symbionts during bleaching is insufficient to counter accelerated photoinactivation. Limnology and Oceanography 56: 139–146.

[71] Hennige SJ, McGinley MP, Grottoli AG, Warner ME (2011) Photoinhibition of *Symbiodinium* spp. within the reef corals *Montastraea faveolata* and *Porites astreoides*: implications for coral bleaching. Marine Biology 158: 2515–2526.

[72] Al-Horani FA, Al-Moghabri S, De Beer D (2003) Microsensor study of photosynthesis and calcification in the scleractinian coral, *Galaxea fascicularis*: active internal carbon cycle. Journal of Experimental Marine Biology and Ecology 288: 1–15.

[73] Lesser MP (2011) Coral bleaching: causes and mechanisms. In: Stambler N, editor.Coral reefs: an ecosystem in transition.New York: Springer Press. pp. 405–419.

[74] Ragni M, Airs RL, Hennige SJ, Suggett DJ, Warner ME, et al (2010) PSII photoinhibition and photorepair in *Symbiodinium* (Pyrrhophyta) differs between thermally tolerant sensitive phylotypes. Marine Ecology Progress Series 406: 57–70.

[75] Ulstrup KE, Ralph PJ, Larkum AWD, Kühl M (2006) Intra-colonial variability in light acclimation of zooxanthellae in coral tissues of *Pocillopora damicornis* . Marine Biology 149: 1325–1335.

[76] Parys E, Jastz H (2006) Light-enhanced dark respiration in leaves, isolated cells and protoplasts of various types of C4 plants. Journal of Plant Physiology 163: 638–647. 16545997 10.1016/j.jplph.2005.05.009

[77] Crawley A, Kline DI, Dunn S, Anthony KRN, Dove S (2010) The effect of ocean acidification on symbiont photorespiration and productivity in *Acropora formosa* . Global Change Biology 16: 851–863.

[78] Schreiber U, Hormann H, Asada K, Neubauer C (1995) O_2_-dependent electron flow in intact spinach chloroplasts: properties and possible regulation of the Mehler-Ascorbate-Peroxidase cycle. In: Mathis P, editor.Photosynthesis: from Light to Biosphere.Dordrecht, The Netherlands: Kluwer Academic Publishers. pp. 813–818.

[79] Suggett DJ, Warner ME, Smith DJ, Davey P, Hennige S, et al (2008) Photosynthesis and production of hydrogen peroxide by *Symbiodinium* (Pyrrhophyta) phylotypes with different thermal tolerances. Journal of Phycology 44: 948–956. 27041613 10.1111/j.1529-8817.2008.00537.x

[80] Glud RN, Ramsing NB, Revsbech NP (1992) Photosynthesis and photosynthesis-coupled respiration in natural biofilms quantified with oxygen microsensors. Journal of Phycology 28: 51–60.

[81] Raven JA (1992) Biology of Plants. New York: Worth Publisher Inc.

