# Supplementary material for: Light Respiratory Processes and Gross Photosynthesis in Two Scleractinian Corals
Source: PLoS One. 2014 Oct 31;9(10):e110814. doi: 10.1371/journal.pone.0110814 (PMC4216011; doi:10.1371/journal.pone.0110814)
Supplement: Table S1 — Gas exchange rates measured as a function of irradiance for Pocillopora damicornis and Pavona decussata . Following gas exchange rates are presented: GPO2 micro – In hospite gross O2 production (microsensor based), PnetO2 micro – net photosynthetic O2 production (microsensor based), Rlight O2 micro – light O2 respiration (microsensor based), GCO2 PBR – Gross CO2 exchange for 6 light intensities. (DOCX) [file pone.0110814.s001.docx]

Table S1: Gas exchange rates measured as a function of irradiance for *Pocillopora damicornis* and *Pavona decussata.* Following gas exchange rates are presented: GP_O2 micro_ – *In hospite* gross O_2_ production (microsensor based), Pnet_O2 micro_ – net photosynthetic O_2_ production (microsensor based), R_light O2 micro_ – light O_2_ respiration (microsensor based), G_CO2 PBR_ – Gross CO_2_ exchange for 6 light intensities.

| Light intensity | *Pocillopora damicornis* | | | |
| --- | --- | --- | --- | --- |
|  | GP_O2 micro_  (nmol cm^-2^ s^-1^) | Pnet _O2 micro_  (nmol cm^-2^ s^-1^) | R_light O2 micro_  (nmol cm^-2^ s^-1^) | G_CO2 PBR_  (nmol cm^-2^ s^-1^) |
| 0 | 0.000 ± 0.000 | -0.019 ± 0.003 | 0.019 ± 0.005 | 5.0414 ± 1.3564 |
| 40 | 0.040 ± 0.016 | -0.008 ± 0.008 | 0.048 ± 0.005 | 1.7118 ± 0.2172 |
| 80 | 0.117 ± 0.008 | 0.003 ± 0.003 | 0.114 ± 0.003 | 1.4439 ± 0.1489 |
| 210 | 0.484 ± 0.050 | 0.016 ± 0.002 | 0.468 ± 0.048 | 1.7334 ± 0.3796 |
| 560 | 0.485 ± 0.002 | 0.034 ± 0.013 | 0.451 ± 0.020 | 1.8751 ± 0.4761 |
| 1100 | 0.536 ± 0.044 | 0.029 ± 0.011 | 0.507 ± 0.060 | 1.3489 ± 0.5634 |
|  | | | | |
|  | *Pavona decussata* | | | |
|  | GP_O2 micro_  (nmol cm^-2^ s^-1^) | Pnet _O2 micro_  (nmol cm^-2^ s^-1^) | R_light O2 micro_  (nmol cm^-2^ s^-1^) | G_CO2 PBR_  (nmol cm^-2^ s^-1^) |
| 0 | 0.000 ± 0.000 | -0.027 ± 0.013 | 0.027 ± 0.013 | 1.3804 ± 0.4882 |
| 40 | 0.097 ± 0.026 | -0.016 ± 0.006 | 0114 ± 0.020 | 1.1675 ± 0.1384 |
| 80 | 0.153 ± 0.015 | 0.002 ± 0.003 | 0.151 ± 0.012 | 2.2600 ± 0.7541 |
| 210 | 0.346 ± 0.084 | 0.043 ± 0.003 | 0.303 ± 0.081 | 2.8885 ± 0.5974 |
| 560 | 0.546 ± 0.104 | 0.038 ± 0.014 | 0.508 ± 0.118 | 2.7749 ± 0.4079 |
| 1100 | 0.507 ± 0.066 | 0.023 ± 0.016 | 0.484 ± 0.050 | 2.4551 ± 0.5517 |
